# Editorial: Advancing the fight against bone and joint infections: a special issue in diagnostics and treatment

**DOI:** 10.3389/fsurg.2023.1228265

**Published:** 2023-06-06

**Authors:** Markus Rupp, Mustafa Citak, Irene Katharina Sigmund

**Affiliations:** ^1^ Department of Trauma Surgery, University Hospital Regensburg, Regensburg, Germany; ^2^ Helios ENDO-Klinik Hamburg, Hamburg, Germany; ^3^ Nuffield Orthopaedic Centre, Oxford University Hospitals NHS Foundation Trust, Oxford, United Kingdom

**Keywords:** osteomyelitis, periprosthetic joint infection, spondylodiscitis, implant associated infection, bone infection

**Editorial on the Research Topic** Diagnostics and treatment for bone and joint infections

In the relentless pursuit of medical progress, researchers and healthcare professionals are continually striving to improve diagnostics and treatment strategies for bone and joint infections. We are delighted to present a special issue in Frontiers in Surgery, dedicated to the crucial theme of “Diagnostics and Treatment of Bone and Joint Infections.” This collection of manuscripts brings together a wealth of knowledge and expertise, shedding light on various aspects of these challenging infections. We extend our heartfelt gratitude to the authors, reviewers, and the dedicated editorial team who have contributed to the realization of this remarkable compilation.

First and foremost, we express our sincere appreciation to the authors who have passionately devoted their time and expertise to share their valuable research findings. Their commitment to advancing our understanding of bone and joint infections has brought forth groundbreaking contributions to this special issue. Without their dedication, the wealth of knowledge presented within these manuscripts would not have been possible. We would also like to extend our deepest gratitude to the reviewers who diligently provided their time, expertise, and constructive feedback. Their meticulous evaluation and insightful suggestions have played an invaluable role in ensuring the high quality and scientific rigor of the manuscripts in this special issue. Furthermore, we extend our heartfelt thanks to the editorial team, whose unwavering support and guidance have been instrumental in the success of this special issue. Their commitment to maintaining the highest standards of scholarly publication, along with their astute management and organization, has facilitated the realization of this ambitious endeavor.

Within this special issue, several noteworthy manuscripts have made significant contributions to the field of bone and joint infection research. The manuscript titled “Symptom Duration is Associated with Failure of Periprosthetic Joint Infection Treated with Debridement, Antibiotics, and Implant Retention” provides crucial insights into prognostic factors in periprosthetic joint infections. Similarly, the manuscript titled “Treatment of Periprosthetic Joint Infection and Fracture-Related Infection with a Temporary Arthrodesis Made by PMMA-Coated Intramedullary Nails – Evaluation of Technique and Quality of Life in Implant-Free Interval” explores an innovative treatment approach and its impact on patients' quality of life.

In addition, the manuscript titled “Development and Validation of a Diagnostic Model for Differentiating Tuberculous Spondylitis from Brucellar Spondylitis Using Machine Learning: A Retrospective Cohort Study” demonstrates the potential of machine learning in differentiating between two challenging forms of spondylitis. The manuscript titled “Risk Factors for Tuberculous or Nontuberculous Spondylitis After Percutaneous Vertebroplasty or Kyphoplasty in Patients with Osteoporotic Vertebral Compression Fracture: A Case-Control Study” highlights crucial risk factors associated with spondylitis following spinal procedures. Moreover, the manuscript titled “Therapy of Chronic Extensor Mechanism Deficiency After Total Knee Arthroplasty Using a Monofilament Polypropylene Mesh” offers innovative therapeutic approaches for addressing complications after knee arthroplasty. Additionally, the manuscripts discussing the use of D-lactate as a biomarker for periprosthetic joint infection, the C-reactive protein to lymphocyte ratio as a predictor of surgical site infection after posterior lumbar interbody fusion and instrumentation, and the impact of time to reimplantation on reinfection risk in two-stage revision for periprosthetic infection provide valuable insights into diagnosis and treatment strategies.

The culmination of these manuscripts serves as a testament to the relentless pursuit of researchers and healthcare professionals in combating bone and joint infections. Their tireless efforts, dedication, and enthusiasm to prevent and effectively treat these devastating types of infections are commendable. Their research not only enhances our understanding but also provides hope for improved outcomes, enhanced patient care, and a future where bone and joint infections are conquered.

In conclusion, we express our sincere gratitude to all the researchers, doctors, and healthcare professionals who have contributed to this special issue. Their collective efforts bring us closer to our shared goal of combating bone and joint infections, alleviating patient suffering, and improving overall healthcare outcomes. Together, let us continue the journey towards a future free from the devastating impact of these infections.

